# Proximity-dependent Mapping of the Androgen Receptor Identifies Kruppel-like Factor 4 as a Functional Partner

**DOI:** 10.1016/j.mcpro.2021.100064

**Published:** 2021-02-26

**Authors:** Lauriane Vélot, Frédéric Lessard, Félix-Antoine Bérubé-Simard, Christophe Tav, Bertrand Neveu, Valentine Teyssier, Imène Boudaoud, Ugo Dionne, Noémie Lavoie, Steve Bilodeau, Frédéric Pouliot, Nicolas Bisson

**Affiliations:** 1 Centre de recherche du Centre Hospitalier Universitaire (CHU) de Québec-Université Laval, Axe Oncologie, Québec, Quebec, Canada; 2 Centre de recherche sur le cancer de l’Université Laval, Québec, Quebec, Canada; 3 PROTEO-Quebec Network for Research on Protein Function, Engineering, and Applications, Québec, Quebec, Canada; 4 Centre de recherche en données massives de l’Université Laval, Québec, Québec, Canada; 5 Department of Molecular Biology, Medical Biochemistry and Pathology, Faculté de Médecine, Université Laval, Québec, Quebec, Canada; 6 Department of Surgery, Faculté de Médecine, Université Laval, Québec, Quebec, Canada

**Keywords:** AP-MS, affinity purification combined to mass spectrometry, AR, androgen receptor, ARE, androgen response element, DHT, 5α-dihydrotestosterone, DMAP1, DNA methyltransferase 1 associated protein 1, DNA-PK, DNA-dependent protein kinase, FBS, fetal bovine serum, GFP, green fluorescent protein, GRHL2, grainyhead-like 2, HEK293, human embryonic kidney 293 cells, HSP, heat shock protein, KLF4, Krüppel-like factor 4, KMT2A, lysine methyl transferase 2A, MALDI-TOF, matrix-assisted laser desorption ionization time-of-flight, MED1, MEDiator complex subunit 1, MED12, MEDiator complex subunit 12, NIPBL, NIPBL cohesion loading factor, PCa, prostate cancer, PEI, polyethylenimine, POLH, DNA polymerase eta, PSA, prostate-specific antigen, RIME, rapid immunoprecipitation MS of endogenous proteins, TF, transcription factor

## Abstract

Prostate cancer (PCa) is the most frequently diagnosed cancer in men and the third cause of cancer mortality. PCa initiation and growth are driven by the androgen receptor (AR). The AR is activated by androgens such as testosterone and controls prostatic cell proliferation and survival. Here, we report an AR signaling network generated using BioID proximity labeling proteomics in androgen-dependent LAPC4 cells. We identified 31 AR-associated proteins in nonstimulated cells. Strikingly, the AR signaling network increased to 182 and 200 proteins, upon 24 h or 72 h of androgenic stimulation, respectively, for a total of 267 nonredundant AR-associated candidates. Among the latter group, we identified 213 proteins that were not previously reported in databases. Many of these new AR-associated proteins are involved in DNA metabolism, RNA processing, and RNA polymerase II transcription. Moreover, we identified 44 transcription factors, including the Kru¨ppel-like factor 4 (KLF4), which were found interacting in androgen-stimulated cells. Interestingly, KLF4 repressed the well-characterized AR-dependent transcription of the *KLK3* (*PSA*) gene; AR and KLF4 also colocalized genome-wide. Taken together, our data report an expanded high-confidence proximity network for AR, which will be instrumental to further dissect the molecular mechanisms underlying androgen signaling in PCa cells.

## Highlights

• BioID proteomics identifies 267 androgen receptor (AR)-associated candidates
• Krüppel-like factor 4 (KLF4) is a new AR interaction partner
• AR and KLF4 colocalize genome-wide on >4000 genes, including *KLK3* (*PSA*)
• KLF4 acts as a repressor for the AR target gene *KLK3* (*PSA*)

## Graphical Abstract

**Figure:**
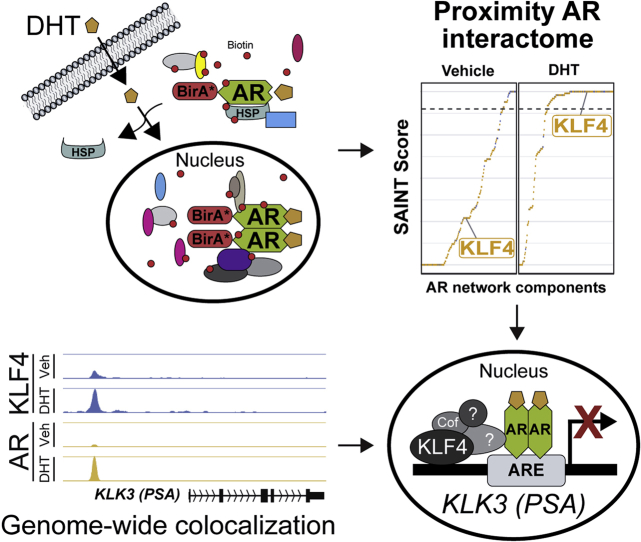

## In Brief

A proximity interaction network for the androgen receptor (AR) was obtained from androgen-responsive prostate cancer cells. A total of 267 candidates were identified, most associating following ligand stimulation, including Krüppel-like factor 4 (KLF4). KLF4 and AR were found to colocalize genome-wide on 4097 genes including *PSA* (*KLK3*), for which KLF4 acts as a repressor, without regulating the expression of AR. These results are instrumental to further dissect the molecular mechanisms underlying androgen signaling in prostate cells.

Prostate cancer (PCa) is the most frequently diagnosed cancer in men and is the third cause of cancer mortality (1). PCa initiation and growth are driven by the androgen receptor (NR3C4, AR), a steroid receptor that belongs to the nuclear receptor family (2). Following binding by androgens such as dihydrotestosterone (DHT) in the cytoplasm, AR undergoes a conformational change, which allows its release from heat shock chaperone proteins (HSPs) and the formation of phosphorylated AR homodimers (3, 4). AR is then able to translocate to the nucleus where it binds DNA on androgen response elements (AREs). As a transcription factor (TF), AR regulates along with cofactors the transcription of target genes involved in proliferation, survival, and cell growth or acts as a cofactor for other TFs (5, 6). Its best characterized target gene is *Kallikrein-Related Peptidase 3* (*KLK3*), often referred to as *Prostate-Specific Antigen* (*PSA*). Moreover, AR is phosphorylated and activated in a ligand-independent manner by kinases such as MAPK and PI3K/AKT (7, 8). Thus, AR function is regulated by a number of proteins that affect ligand binding, protein folding, nuclear translocation, and transcriptional activation. However, the extent of these AR-associated proteins is still poorly understood.

Several studies shed light on AR protein interaction networks using unbiased approaches such as affinity purification combined to mass spectrometry (AP-MS). Using matrix-assisted laser desorption ionization time-of-flight (MALDI-TOF) MS on purified AR complexes, Ishitani *et al.* identified a number of associated proteins including RNA-binding protein P54NRB/NONO (9). They further demonstrated that it functions as a transcriptional coactivator for AR. In another report, Faus *et al.* utilized DNA corresponding to ARE-2 as bait to characterize AR complexes using MS and identified the ubiquitin-specific protease USP10 (10). Mayeur *et al.* performed pull-downs using GST-tagged AR N-terminal or C-terminal polypeptides combined to MS to delineate an association between AR and the DNA-dependent protein kinase (DNA-PK) complex (11). Likewise, Chen *et al.* utilized AR AP-MS to discover an androgen-dependent association between endogenous AR and the deubiquitinating enzyme USP7 (12). More recently, a few groups reported the identification of AR-associated proteins at a larger scale. Paltoglou *et al.* took advantage of the RIME method (rapid immunoprecipitation MS of endogenous proteins) to identify 54 and 75 candidates with wild-type AR and the constitutively active ARv567es variant, respectively. Interestingly, they identified the transcription factor Grainyhead-like 2 (GRHL2) and characterized its function as a coregulator of AR, both as an oncogenic enhancer of androgen signaling and as a suppressor of metastasis (13). In another study, Stelloo *et al.* also made use of RIME and reported 66 proteins in the AR interaction network in LNCaP cells. They further validated some of these interactions in LAPC4 cells and in prostate cancer patient-derived xenograft models (14). Hsiao *et al.* took advantage of cellular fractionation to determine the cytosolic AR protein interactome and to identify proteins implicated in androgen-dependent AR-mediated gene transcription (15, 16). Paliouras *et al.* used three AR genetic variants and delineated their protein interaction network to help predict prostate cancer clinical outcome (17). While these AP-based MS approaches have been instrumental for the characterization of AR protein interaction networks, they have often come short of identifying proteins displaying weaker or more transient interactions due to technical limitations (18).

To circumvent this, Roux *et al.* developed proximity labeling proteomics, namely BioID (19), a method based on the fusion of a mutant biotin ligase BirA∗ with a protein of interest. Upon addition of biotin to the culture medium, the promiscuous BirA∗ chimera will favor covalent biotin binding to adjacent proteins, which may be affinity-purified and identified by MS. Lempiainen *et al.* used this approach to define a proximity network for either glucocorticoid receptor and AR overexpressed in DHT-stimulated human embryonic kidney (HEK) 293 cells (20), which do not endogenously express detectable levels of AR (21). A total of 32 DHT-dependent high-confidence proximity interactions were identified. Among this group, ten were previously reported in the BioGRID, GPS-Prot, or IntAct databases (22, 23, 24), including NCOR1 (25), JMJD1C (26), SMARCA4/BRG1 and SMARCD1/BAF60a (27), and TCF20/SPBP (28).

Here, we performed BioID proximity labeling proteomics in androgen-dependent LAPC4 cells that express wild-type AR. We delineate an AR proximity network containing 267 proteins, most of which associated following ligand stimulation, providing a large high-confidence interaction network for AR obtained from androgen-responsive cells. Among the AR-associated proteins, we reveal 213 candidates that were not previously reported in the BioGRID, GSP-Prot, or IntAct database and describe the identification of the Krüppel-like factor 4 (KLF4) as a new AR-associated protein. Finally, we show that KLF4 and AR colocalize genome-wide on a number of genes including PSA (*KLK3*) and surprisingly act as a repressor for the latter, without regulating the expression of *AR* itself.

## Experimental Procedures

### Plasmid Constructions and Adenoviral Production

Human AR (NCBI clone NP_000035.2) was subcloned into pMSCVpuro (Clontech) in fusion with an N-terminal 3xFLAG epitope tag and a BirA∗ sequence. pEGFP-N1 was obtained from Clontech and human AR was subcloned into pEGFP-C1 (Clontech) in fusion with a N-terminal GFP protein tag. Human KLF4 (NCBI clone NP004226.3) was subcloned into pMSCVpuro (Clontech) with an N-terminal 3xFLAG tag. All inserts were fully sequenced and protein expression was verified by western blot. Adenoviral plasmids for PSEBC-TSTA were previously described (29) and transfected into HEK293T  cells for adenovirus production. Titers were determined using the Adeno-X Rapid Titer Kit (Clontech, Mountain View, CA, USA).

The DsiRNA catalog numbers and sequences were as follows (Integrated DNA Technologies, Coralville IA, USA): DsiAR (HSC.RNAI.N000044.12.1_1 nm; #134941205 and HSC.RNAI.N000044.12.8_2 nm; #134941208), DsiKLF4 (hs.Ri.KLF4.13.1; #153062069 and hs.Ri.KLF4.13.2; #153062072), DsiGRHL2 (hs.Ri.GRHL2.13.2; #153062024 and hs.Ri.GRHL2.13.3; #153062027), DsiMAML (hs.Ri.MAML1.13.1; #153062066 and hs.Ri.MAML1.13.2; #153062076), DsiRBPJ (hs.Ri.RBPJ.13.2; #153062030 and hs.Ri.RBPJ.13.3; #153062033), and DsiTBL1X (hs.Ri.TBL1X.13.1; #153062060 and hs.Ri.TBL1X.13.2; #153062063).

**Table:** 

Targets	Reverse sequence	Forward sequence
siAR#1	rGrCrCrUrUrUrArArArUrCrUrGrUrGrArUrGrArUrCrCrUCA	rUrGrArGrGrArUrCrArUrCrArCrArGrArUrUrUrArArArGrGrCrArU
siAR#2	rCrUrGrUrUrArUrArArCrUrCrUrGrCrArCrUrArCrUrCrCTC	rGrArGrGrArGrUrArGrUrGrCrArGrArGrUrUrArUrArArCrArGrGrC
siKLF4#1	rArGrCrArCrUrArCrArArUrCrArUrGrGrUrCrArArGrUrUCC	rGrGrArArCrUrUrGrArCrCrArUrGrArUrUrGrUrArGrUrGrCrUrUrU
siKLF4#2	rGrUrUrCrUrArArArGrGrUrArCrCrArArArCrArArGrGrAAG	rCrUrUrCrCrUrUrGrUrUrUrGrGrUrArCrCrUrUrUrArGrArArCrCrA
siGRHL2#1	rGrArGrCrUrUrUrArArUrArCrGrArUrUrGrGrArArArCrATT	rArArUrGrUrUrUrCrCrArArUrCrGrUrArUrUrArArArGrCrUrCrUrC
siGRHL2#2	rUrGrUrCrArUrCrUrUrGrGrArArUrUrGrGrUrUrUrCrUrAAA	rUrUrUrArGrArArArCrCrArArUrUrCrCrArArGrArUrGrArCrArUrC
siMAML1#1	rCrUrGrUrUrGrArArArCrUrUrUrArGrArUrArGrCrArGrAAT	rArUrUrCrUrGrCrUrArUrCrUrArArArGrUrUrUrCrArArCrArGrArA
siMAML1#2	rCrGrCrArUrCrUrUrCrArUrGrArUrArCrArGrUrUrArArGAG	rCrUrCrUrUrArArCrUrGrUrArUrCrArUrGrArArGrArUrGrCrGrUrG
siRBPJ#1	rGrCrArUrUrUrUrArCrCrUrUrArArGrGrArUrArCrArGrAAA	rUrUrUrCrUrGrUrArUrCrCrUrUrArArGrGrUrArArArArUrGrCrArC
siRBPJ#2	rGrCrArUrGrCrUrCrUrArCrGrCrArUrUrCrArGrUrCrCrUTA	rUrArArGrGrArCrUrGrArArUrGrCrGrUrArGrArGrCrArUrGrCrUrG
siTBL1X#1	rCrArUrUrUrGrUrUrUrCrArArGrArGrArGrArArUrCrArACA	rUrGrUrUrGrArUrUrCrUrCrUrCrUrUrGrArArArCrArArArUrGrArG
siTBL1X#2	rUrCrArGrUrCrArArUrArArUrCrArCrGrCrGrArArGrCrCAA	rUrUrGrGrCrUrUrCrGrCrGrUrGrArUrUrArUrUrGrArCrUrGrArArU

Nontargeting DsiRNAs provided by the manufacturer were used as negative controls.

### Cell Culture and Transfection

LNCaP and VCaP cells were obtained from ATCC. LAPC4 cells were kindly provided by Dr C. Sawyers. LNCaP were cultured in RPMI 1640 media containing 10% fetal bovine serum (FBS), LAPC4 and VCaP cells were cultured in DMEM media containing 10% FBS. Cell lines were tested for *mycoplasma* using the MycoAlert *Mycoplasma* Detection kit (Lonza, Basel, Switzerland). Transfections were performed using Lipofectamine 2000 (Thermo Fisher Scientific). Stable LAPC4 cells were selected with puromycin (5 μg/ml) for 1 month. Individual clones were picked and selected based on transgene expression. DsiRNAs (5 nM) were transfected using Lipofectamine 2000 (Thermo Fisher) following manufacturer’s guidelines.

### Cell Lysis and BioID Proximity Labeling

For BioID experiments, LAPC4 cells were seeded at 50% confluence in four 15 cm tissue culture dishes per condition and grown for 24 h prior to treatments. Cells were treated with DHT (10 nM) for 0 h, 24 h, or 72 h. Biotin (50 μM) was added for the last 24 h prior to cell lysis. Cells were washed twice with PBS, then scrapped in PBS buffer before lysis in ice-cold RIPA buffer followed by benzonase (Sigma-Aldrich) treatment for 1 h and three cycles of sonication. Protein concentration was measured and normalized using a BCA kit (Thermo Fisher Scientific). Lysates were incubated under agitation for 3 h at +4 °C with 30 μl streptavidin agarose beads (Sigma-Aldrich). Beads were washed three times with lysis buffer. For MS experiments, beads were additionally washed twice with 20 mM Tris pH 7.4, and proteins were eluted by incubation with agitation at +4  °C with 50 mM H_3_PO_4_ before digestion with trypsin, as described (30). A stage-tip purification step through a C18 column was performed in order to desalt further protein samples (31). Proteins were eluted in 0.5% acetic acid: 80% acetonitrile.

For western blotting experiments, cells were washed once with PBS and lysed in 1x Laemmli buffer. Whole-cell lysates were normalized based on their protein concentration, as assessed by BCA assay.

### Affinity Purification

A total of 5 x 10^6^ HEK293T cells were seeded in 10 cm tissue culture dishes and grown for 24 h. Cells were transiently transfected using polyethylenimine (PEI) with pEGFP-N1 or pEGFP-C1-AR to achieve similar levels of purification for GFP or GFP-AR and cotransfected with pMSCV(puro)-3xFLAG-KLF4. Cells were scraped into IP buffer (50 mM Tris-HCl, pH 7.9, 1 mM EDTA, 0.1 mM EGTA, 12.5 mM MgCl_2_, 400 mM NaCl, 20% glycerol, 1% Triton X-100 (BioShop, Burlington, ON) containing protease (P8340, Sigma-Aldrich) and phosphatase inhibitors (Cocktail 2, Sigma-Aldrich). Cell lysates were kept on ice for 15 min and then sonicated 30 s at a low intensity. Cell lysates were cleared by centrifugation at 20,000 *g* for 1 min, and purifications were performed with 5 μl of GFP-Trap Agarose beads (Chromotek, New York) for 2h at +4^o^C. Beads were washed twice with IP buffer. Precipitates were recovered after 2 h of incubation on a rotating machine at +4^o^C and washed three times 10 min in IP buffer. Precipitates and total cell lysates were separated by SDS-PAGE and transferred to nitrocellulose membranes prior to western blotting with the indicated antibodies.

### Western Blotting and Antibodies

A total of 10–20 μg of whole-cell lysates was resolved by SDS-PAGE and transferred to nitrocellulose membranes (GE Healthcare). Loading of each track was verified with Ponceau S (Sigma-Aldrich) staining. Antibodies used were as follows: mouse anti-AR (Santa Cruz, #7305), rabbit anti-AR (Santa Cruz, #816), rabbit anti-KLF4 (Abcam, #215036), mouse anti-tubulin (Cell Signaling Technology, #3873), mouse anti-actin (Cell Signaling Technology, #3700), anti-GFP (Abcam, #290), rabbit anti-MED1 (Bethyl, #A300–793A), rabbit anti-MED12 (Bethyl, #A300–774A), rabbit anti-KMT2A (Bethyl, #A300–087A), mouse anti-DMAP1 (Santa Cruz, #373949), rabbit anti-POLH (Bethyl, #A301–231A), rabbit anti-NIPBL (Bethyl, #A301–779A), or mouse H3 (Cell Signaling Technology, #3638). Secondary antibodies were the following: anti-FLAG M2 (Sigma-Aldrich), horse anti-mouse HRP-linked IgG (Cell Signaling Technology, #7076), goat anti-rabbit HRP-linked IgG (Cell Signaling Technology, #7074), or streptavidin-HRP (Life Technologies #434323). Signal was revealed using BioRad Clarity Western ECL substrate and detected either on Hyblot CL autoradiography films (Denville) or with an Amersham Imager 600RGB (GE Healthcare). Signal quantification was performed using Image J software gel analysis tools (NIH).

### Luciferase Assay

LAPC4 (1.0 x 10^5^ cells/well), LNCaP (1.0 x 10^5^ cells/well), and VCaP (1.6 x 10^5^ cells/well) were seeded in 24-well plates with media containing 5% charcoal stripped FBS and grown for 18 h before PSEBC-TSTA (3.5 MOI) adenovirus infection, DsiRNA transfection, and treatment with vehicle (EtOH) or DHT (10 nM). Seventy-two hours following infection, cells were washed once with HBSS and lysed. Luciferase assay was performed following manufacturer guidelines (Promega) with 20 uL of each lysate. Relative luminescence unit (RLU) was normalized by protein content in each well (normalized RLU = total RLU/total protein amount). Protein concentration was estimated by adding 250 μl of Bradford reagent (ThermoFisher Scientific) to 3 μl of total lysate. Absorbance was measured using an Infinite F50 absorbance microplate reader (Tecan, Mannedorf, Switzerland) at 595 nm.

### RT-qPCR Assay

LAPC4 (6 x 10^5^ cells/well) cells were seeded in 6-well plates with DMEM medium containing 5% charcoal stripped FBS, transfected with DsiRNAs, and grown 24 h before vehicle/DHT treatment. Cells were washed twice with PBS, and total RNA was extracted using TriPure reagent (Sigma), according to the manufacturer’s recommendations. RNAs were cleaned using the GeneJET RNA purification kit (Thermo Fisher Scientific). Reverse transcription was performed using 2.5 μg of total RNA according to the standard SuperScript VILO Master Mix (Invitrogen). The resulting cDNAs were diluted at 1/24 and then 10 μL was used in a quantitative PCR reaction carried out with the SYBR Select Master Mix (Applied Biosystems). *Actin* was used for normalization of RT-qPCR data. Fold changes in mRNA expression levels were calculated using the comparative Ct method.

The following primers were used:

**Table:** 

Targets	Reverse sequence	Forward sequence
AR	CTGATGCAGCTCTCTCGC	CCCACATCCTGCTCAAGACG
KLF4	GGGCCCAATTACCCATCCTT	GGCATGAGCTCTTGGTAATGG
PSA	CCTCACAGCTACCCACTGCA	GATGAAACAGGCTGTGCCG
Actin	GCCCACATAGGAATCCTTCTGAC	AGGCACCAGGGCGTGAT

### Immunofluorescence and Microscopy

LNCaP cells were starved overnight, then transfected with DsiRNAs for 48 h. Cells were fixed in ice-cold methanol for 4 min at –20 °C after 4 h of stimulation with DHT (10 nM). This was followed by three washes with PBS and incubation with the following antibodies diluted in blocking buffer (0.2% BSA (Bioshop), 0.1% Triton X100 (Sigma-Aldrich)): rabbit anti-FLAG (Sigma Aldrich, #F7425), mouse anti-AR (Santa Cruz, #7305), or rabbit anti-KLF4 (Abcam, #215036) for 1 h at room temperature. After washes in PBS, coverslips were incubated with Alexa 568-conjugated goat anti-rabbit (Thermo Fisher Scientific, #A11011) or Alexa 488-conjugated goat anti-mouse (Cell Signalling Technology, #4408) antibodies for 1 h at room temperature. They were washed twice with PBS before being mounted on slides using ProLong Gold antifade with DAPI (Thermo Fisher). Pictures were acquired with an Olympus FV1000 using the FluoView 3.0 software or with a Nikon Eclipse E600 imaging system using MetaView.

### Experimental Design and Statistical Rationale

Each BioID experiment was performed in biological triplicate. Controls for each experiment were treated concomitantly to experimental samples. 3xFLAG-BirA∗ with and without biotin and 3xFLAG-BirA∗-AR without biotin were used as controls. The highest total spectra number from any of the three controls was utilized as the control value for SAINT analysis. Biological triplicates were required and sufficient to perform SAINT analyses to distinguish background from *bona fide* protein associations (32). Statistical analyses were performed via two-way ANOVA using Prism version 7 (GraphPad software inc California, USA). *p* values <0.05 were considered significant.

### Mass Spectrometry

Samples were analyzed by nanoLC/MSMS. For each injection, 1 μg of peptide samples was injected and separated by online reversed-phase (RP) nanoscale capillary liquid chromatography (nanoLC) and analyzed by electrospray mass spectrometry (ESI MS/MS). Experiments were performed with a Dionex UltiMate 3000 nanoRSLC chromatography system (Thermo Fisher Scientific/Dionex Softron GmbH) connected to an Orbitrap Fusion mass spectrometer (Thermo Fisher Scientific) driving with Orbitrap Fusion Tune Application 2.0 and equipped with a nanoelectrospray ion source. Peptides were trapped at 20 μl/min in loading solvent (2% acetonitrile, 0.05% TFA) on a 5 mm x 300 μm C18 pepmap cartridge precolumn (Thermo Fisher Scientific/Dionex Softron GmbH) during 5 min. Then, the precolumn was switched online to a house-made 50 cm x 75 μm internal diameter separation column packed with ReproSil-Pur *C18*-*AQ* 3-μm resin (Dr Maisch HPLC GmbH, Ammerbuch-Entringen), and the peptides were eluted with a linear gradient from 5 to 40% solvent B (A: 0,1% formic acid, B: 80% acetonitrile, 0.1% formic acid) in 60 min at 300 nl/min. Mass spectra were acquired using a data-dependent acquisition mode using Thermo XCalibur software version 3.0.63. Full-scan mass spectra (350–1800 m/z) were acquired in the orbitrap using an AGC target of 4e5, a maximum injection time of 50 ms, and a resolution of 120,000. Internal calibration using lock mass on the m/z 445.12003 siloxane ion was used. Each MS scan was followed by acquisition of fragmentation MSMS spectra of the most intense ions and with a minimum intensity threshold of 5000 for a total cycle time of 3 s (top speed mode). The selected ions were isolated using the quadrupole analyzer in a window of 1.6 m/z and fragmented by higher-energy collision-induced dissociation (HCD) with 35% collision energy. The resulting fragments were detected by the linear ion trap in rapid scan rate with an AGC target of 1e4 and a maximum injection time of 50 ms. Dynamic exclusion of previously fragmented peptides was set for a period of 20 s and a tolerance of 10 ppm.

All MS/MS peak lists (MGF files) were generated using Thermo Proteome Discoverer software (Thermo Fisher Scientific Inc, version 2.1.0). MGF sample files were then analyzed using Mascot (Matrix Science, London, UK; version 2.5.1). Mascot was set up to search the Uniprot Complete Proteome *Homo sapiens* database (92,237 entries, February 2017 release) assuming the digestion enzyme trypsin. Mascot was searched with a fragment ion mass tolerance of 0.6 Da and a parent ion tolerance of 10 ppm. Carbamidomethylation of cysteines was set as fixed modification and oxidation of methionine, deamidation of asparagine and glutamine, and phosphorylation on serine, threonine, and tyrosine were specified as a variable modification. Two missed cleavages were allowed.

Scaffold (version 4.8.1), Proteome Software Inc, Portland, OR) was used to validate MS/MS-based peptide and protein identifications. Proteins/peptides FDR rate was set to 1% or less based on decoy database searching. Protein probabilities were assigned by the Protein Prophet algorithm (33). Proteins that contained similar peptides and could not be differentiated based on MS/MS analysis alone were grouped to satisfy the principles of parsimony. Interaction networks were modeled using Cytoscape v3.1.1.

### Chromatin Immunoprecipitation Coupled with Massively Parallel DNA Sequencing (ChIP-seq)

ChIP-seq experiments were performed as described previously (34, 35, 36). Briefly, 50 million of LAPC4 cells treated for 1h with vehicle (EtOH) or DHT (100 nM) were cross-linked for 10 min with 1% formaldehyde and quenched with 125 mM glycine for 5 min. Cells were then washed with PBS, pelleted, flash frozen, and stored at –80 °C. Sonicated DNA fragments were immunoprecipitated with antibodies directed against KLF4 (R&D Systems, AF3640) and AR (Sigma, EMB Millipore, #06–680). Library preparation and high-throughput sequencing were performed at the next-generation sequencing platform of Centre de Recherche CHU de Québec (CRCHUQ), Québec, Canada. Analysis of raw sequencing reads was performed using the MUGQIC ChIP-Seq pipeline (37). Briefly, reads were trimmed for adaptor sequences using Trimmomatic (38). High-quality reads were aligned to the human reference genome (hg38) with BWA aligner (39). PCR duplicates were removed with picard MarkDuplicates (http://broadinstitute.github.io/picard/). Narrow peaks were called using MACS2 callpeak (40), supplying the sequenced corresponding input DNA as background control. Narrow peaks found in all replicates were used as the list of enriched regions. To generate genomic visualizations, samples from pairs of replicates were pooled. Reads from BAM files were extended to 225 bp and normalized using bins per millions mapped reads (BPM) method and a bin size of 10 bases with bamCoverage function from deepTools (41). BPM (per bin) = number of reads per bin/sum of all reads per bin (in millions). ChIP-Seq heatmaps were generated using computeMatrix and plotHeatmap functions from deepTools. Tracks images were generated using the University of California, Santa Cruz (USCS) Genome Browser (42).

## Results

### BioID Proximity Labeling Identifies Known and Novel AR-associated Proteins

**Fig. 1:**
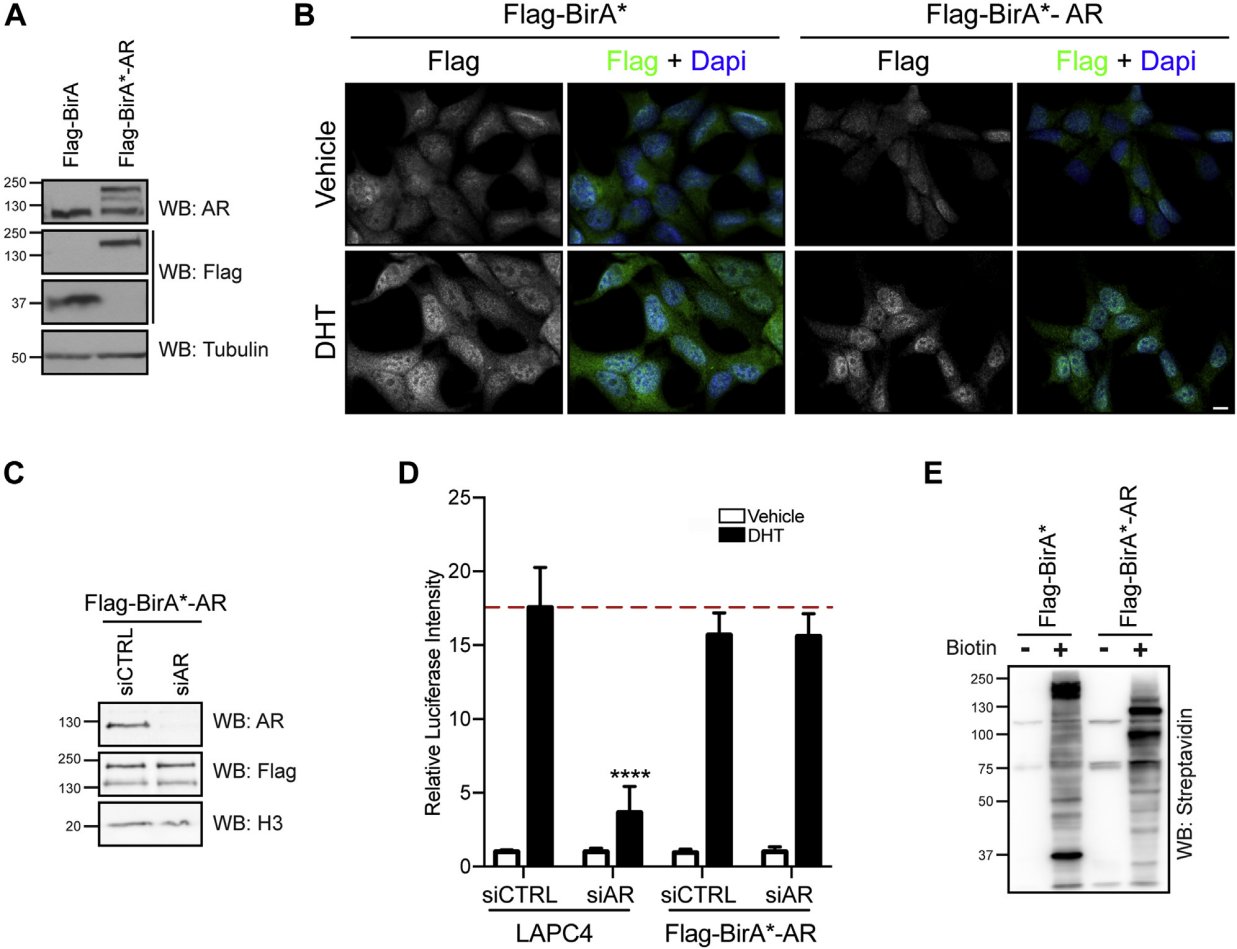
**LAPC4 stable cell lines express a functional Flag-BirA∗-AR chimera.***A*, Clonal LAPC4 stable cell lines expressing either BirA∗ or BirA∗-AR Flag fusion proteins were analyzed by western blot to evaluate transgene expression. *B*, Flag-BirA∗-AR expressing LNCaP cells were analyzed by immunofluorescence in the presence or absence of 10 nM DHT (scale bar: 10 μm). *C*, Flag-BirA∗-AR LAPC4 cells were depleted of endogenous AR using siRNA. Silencing efficiency was assessed by western blot. *D*, Parental (control) and Flag-BirA∗-AR LAPC4 cells were depleted of endogenous AR using siRNA, infected with a luciferase reporter gene coupled with a *PSA* promoter, and treated with a vehicle or 10 nM DHT. Luciferase activity was normalized to the total protein amount for each sample and to the vehicle-treated condition to obtain relative luciferase intensities for each sample. Mean values and standard deviation from three independent experiments are presented. (∗∗∗∗ *p* ≤ 0.0001). *E*, endogenous proteins were biotinylated by Flag-BirA∗ and Flag-BirA∗-AR after 24h of biotin addition to the culture media, as detected by binding to streptavidin. Blots in *A*, *C*, and *E* are representative of three independent experiments.

To establish cell lines relevant to AR function, we selected LAPC4 prostate cells, which express a wild-type AR (43, 44). We generated clonal LAPC4 lines stably expressing AR fused to 3xFLAG-BirA∗ at levels similar to endogenous AR (Fig. 1*A*). To determine whether the BirA∗-AR fusion was functional, we analyzed its subcellular compartmentalization following stimulation with its ligand, DHT. We found that DHT stimulation led to nuclear accumulation of 3xFLAG-BirA∗-AR, as it did for wild-type AR, in LNCaP cells (Fig. 1*B*). To further support this, we tested if 3xFLAG-BirA∗-AR could substitute endogenous AR function in LAPC4 cells. Using a luciferase *PSA* reporter assay (45, 46), we found that 3xFLAG-BirA∗-AR restored transcription in AR-depleted cells (Fig. 1, *C-D*). In addition, we validated that addition of biotin to LAPC4 cells expressing 3xFLAG-BirA∗-AR induced a strong biotinylation pattern (Fig. 1*E*). Together, these observations suggest that 3xFLAG-BirA∗-AR recapitulates endogenous AR functions.

**Fig. 2:**
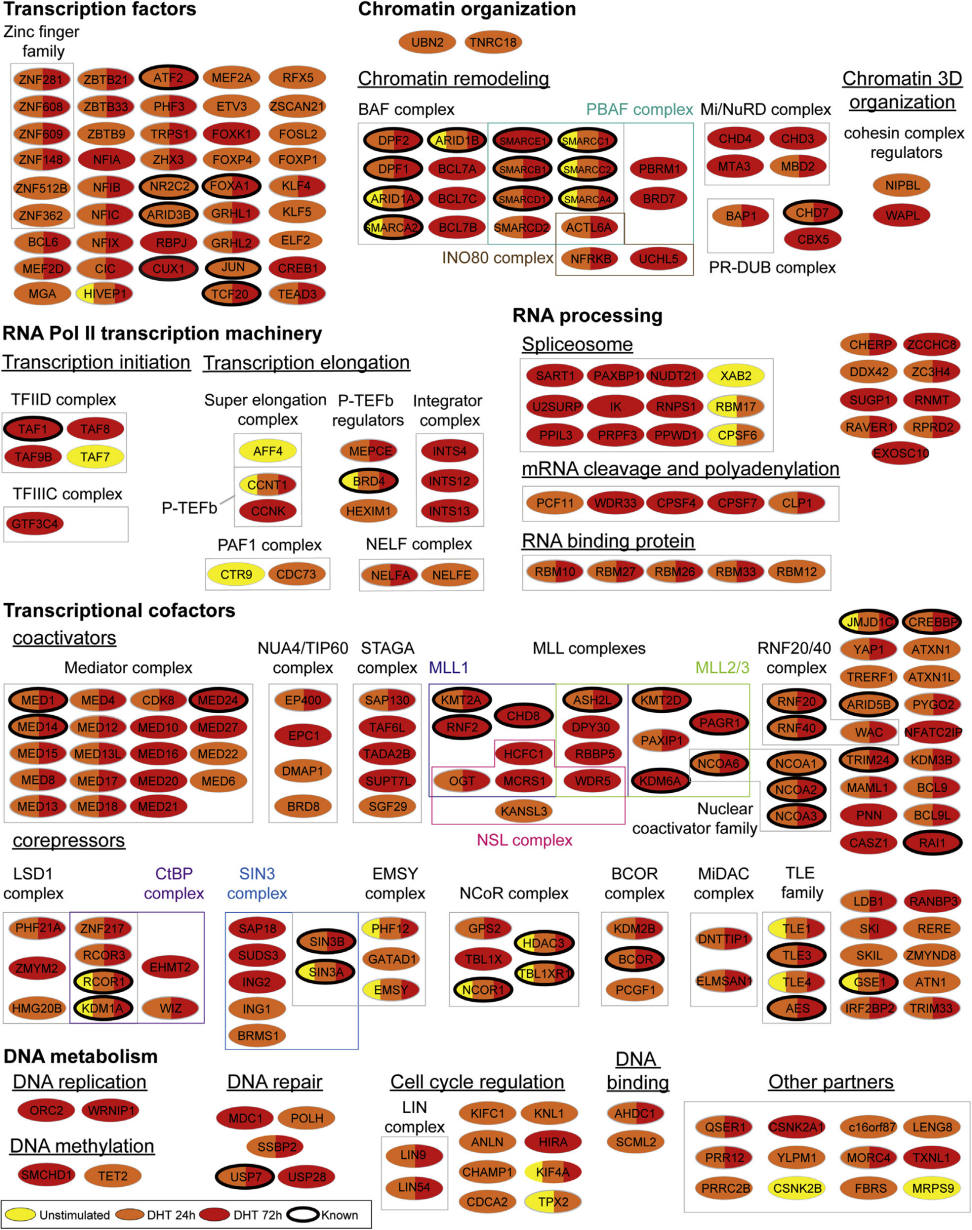
**BioID proximity labeling reveals known and novel AR-associated proteins.** Flag-BirA∗ and Flag-BirA∗-AR expressing cells were stimulated for 24h (*orange*), 72h (*red*) with 10 nM DHT or vehicle (*yellow*) to perform BioID. The AR proximity interaction landscape displays previously reported interactions (known) from the BioGRID, GSP-Prot, and IntAct databases (*circled* in *black*). Proteins were grouped relative to their functions according to CORUM and GeneCards databases.

To delineate the AR proximity network, we affinity-purified biotinylated proteins following the addition of biotin to 3xFLAG-BirA∗-AR and 3xFLAG-BirA∗ LAPC4 cell cultures and identified them using MS. We treated cells with DHT or vehicle for 24h or 72h (47, 48) and performed experiments in biological triplicate. We eliminated nonspecific interactions *via* SAINTexpress (49), using 3xFLAG-BirA∗-expressing cells as controls (supplemental Table S1). Only high-confidence interactions (SAINT score ≤0.9) were considered for follow-up experiments and analyses (supplemental Fig. 1*A*). We identified 31 proteins associated with AR in the absence of agonist (Fig. 2, supplemental Table S1). Strikingly, we also found that 182 and 200 (261 nonredundant) proteins were associated with AR upon stimulation with DHT for 24h or 72h, respectively, despite the loss of six proteins from the 31 detected under nonstimulated conditions. While most of the AR-associated proteins were identified exclusively in DHT-stimulated cells, a few components of the BAF (SMARCA2/4, SMARCC1/2) and NCoR (NCOR1, HDAC3, TBL1XR1) complexes were identified in nonstimulated cells. Among the DHT-dependent associations, we found 54 proteins previously described to associate with AR according to the BioGRID (22), GSP-Prot (23) and IntAct databases (24), including NCOA3/SRC3 (50), SMARCD1 (27), TCF20/SPBP (28) and RNF20 (51). Interestingly, we identified 213 proteins whose association with AR was not reported in databases. We performed affinity purification of GFP or GFP-AR in HEK293 cells and confirmed the interaction between AR and endogenously expressed candidates MEDiator complex subunit 1 (MED1), MEDiator complex subunit 12 (MED12), Lysine MethylTransferase 2A (KMT2A), DNA Methyltransferase 1 Associated Protein 1 (DMAP1), DNA Polymerase eta (POLH), and NIPBL cohesion loading factor (NIPBL) (supplemental Fig. 1, *B–G*). Therefore, this data set meaningfully confirms and expands the number of reported AR-associated proteins according to the BioGRID, GSP-Prot, and IntAct databases.

To depict the extent of our AR proximity network, we manually classified components into groups according to their function (Fig. 2). Strikingly, our analysis revealed a DHT-dependent association between AR and a number of transcription factors. This is consistent with the observation that nuclear receptors may trigger binding of clusters of transcription factors, as shown for the glucocorticoid receptor (52). This analysis also highlighted a number of transcriptional coactivators, including previously characterized RNF20-RNF40 (51), members of the NCoA/SRC family (53, 54), and most components of the mediator complex (supplemental Fig. 1, *B–C*) (55, 56). In addition, we further exposed other transcriptional cofactors and corepressors, as well as components of functional protein complexes involved in chromatin organization (NIPBL (supplemental Fig. 1*G*); BAF, NuRD complexes), transcription initiation (TFIID), cell cycle regulation (LIN complex), or RNA processing (spliceosome) (Fig. 2). Together, the AR proximity network obtained from LAPC4 cells delineates a significant number of known and new AR-associated proteins that play multiple roles in the regulation of gene expression, as well as other cellular processes.

### KLF4 Acts as a Repressor for the AR Target Gene KLK3 (PSA)

**Fig. 3:**
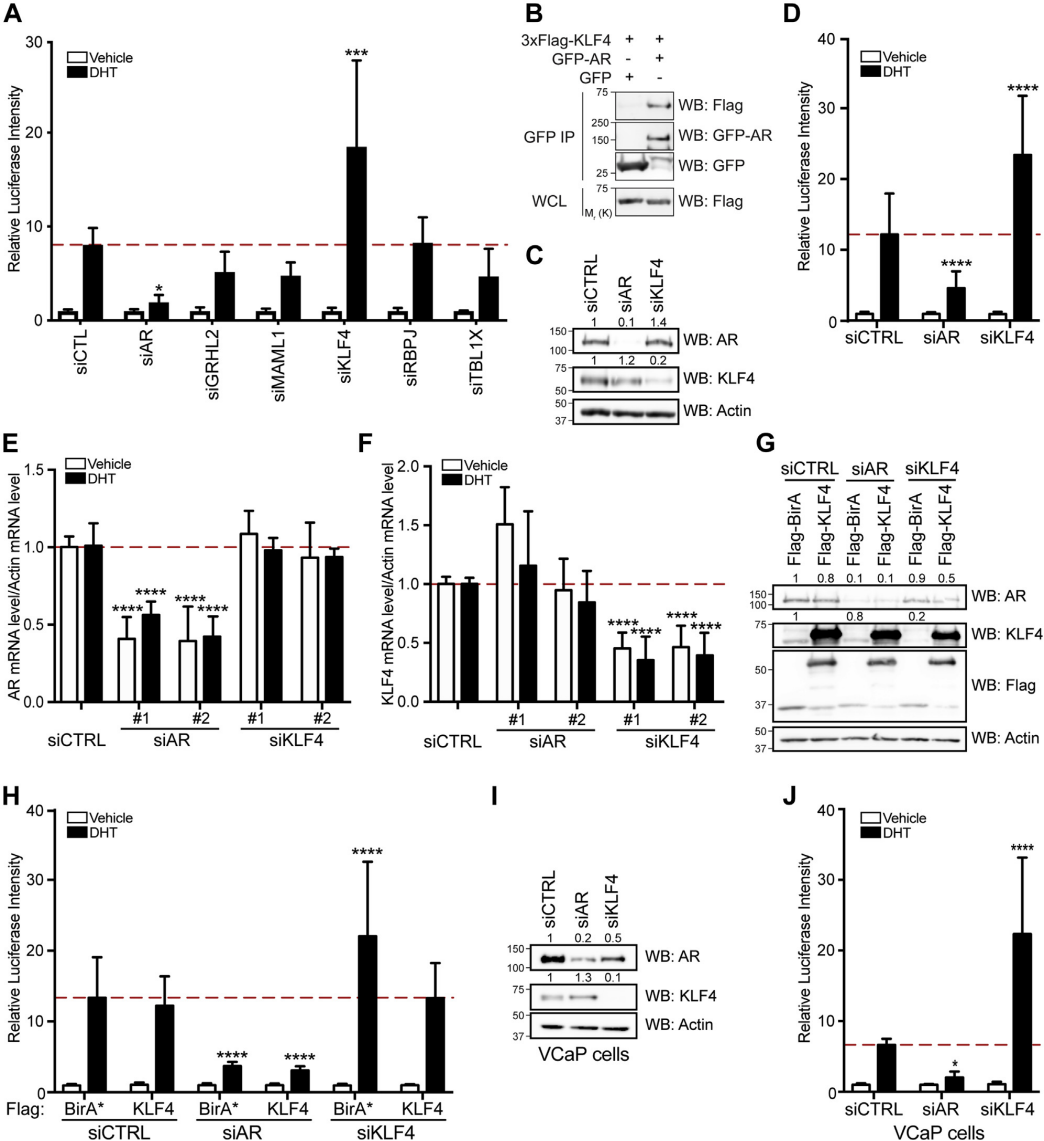
**KLF4 depletion does not affect AR expression but increases its transcriptional activity.***A*, control, siAR, siGRHL2, siMAML1, siKLF4, siRBPJ, and siTBL1X transfected LAPC4 cells were infected with a luciferase reporter gene coupled with a *PSA* promoter and treated with a vehicle or 10 nM DHT. Luciferase activity was normalized to the total protein amount for each sample and to the vehicle-treated condition to obtain relative luciferase intensities for each sample (∗*p* ≤ 0.05; ∗∗∗ *p* ≤ 0.001). *B*, western blot analysis of 3xFLAG-KLF4 following GFP affinity purification in HEK293 T cells cotransfected with GFP/GFP-AR and 3xFLAG-KLF4. *C–D*, Control, siAR, or siKLF4 transfected LAPC4 cells were infected with a luciferase reporter gene coupled with a *PSA* promoter and treated with a vehicle or 10 nM DHT. Normalization was performed as in (*A*). Endogenous AR- or KLF4-depleted cells were analyzed by western blot (*C*) to confirm protein depletion. *E* and *F*, Control, siAR, or siKLF4 transfected LAPC4 cells were stimulated with a vehicle or 10 nM DHT, and RT-qPCR was performed to assess AR (*E*) and KLF4 (*F*) mRNA levels. Data was normalized to actin mRNA levels, and then to the control condition to obtain relative values. *G* and *H*, Control, siAR, or siKLF4 transfected LAPC4 cells were infected with a luciferase reporter gene coupled with a *PSA* promoter, transfected with Flag-BirA or Flag-KLF4, and treated with a vehicle or 10 nM DHT. Endogenous AR- or KLF4-depleted LAPC4 cells were analyzed by western blot (*G*) to determine protein levels. Luciferase activity was normalized to the total protein amount for each sample and to the vehicle-treated condition to obtain relative luciferase intensities for each sample (*H*). *I* and *J*, control, siAR, or siKLF4 transfected VCaP cells were infected with a luciferase reporter gene coupled with a *PSA* promoter and treated with a vehicle or 10 nM DHT. Endogenous AR- or KLF4-depleted cells were analyzed by western blot (*I*) to confirm protein depletion. Luciferase activity was normalized to the total protein amount for each sample and to the vehicle-treated condition to obtain relative luciferase intensities for each sample (*J*). *D, E, F, H, J*, Mean values and standard deviation from three independent experiments are presented (∗*p* ≤ 0.05; ∗∗∗∗ *p* ≤ 0.0001). Blots in *B*, *C*, and *G* are representative of three independent experiments, blots in *I* are representative of two independent experiments.

To investigate the function of these new AR-associated proteins, we selected five candidates (*i.e.*, GRHL2, KLF4, MAML1, RBPJ, TBL1X) based on SAINT score and gene expression in prostate (supplemental Table S1) (57). Using the luciferase *PSA* reporter assay, we tested whether their depletion in LAPC4 cells led to changes in AR-dependent transcriptional regulation. As expected, cells depleted of AR showed a significant decrease in luciferase signal following DHT stimulation relative to control cells (*p* ≤ 0.05, two-way ANOVA), thus confirming the validity of our assay (Fig. 3*A*). KLF4 depletion surprisingly led to a stronger activation of the AR-driven luciferase reporter in DHT-stimulated LAPC4 cells (Fig. 3*A*). While the depletion of the four other AR-associated candidates that we tested did not lead to statistically significant changes, it did induce in most cases a slight decrease in AR reporter activation. We confirmed by coexpression and affinity purification that KLF4 associates with AR (Fig. 3*B*), reinforcing our BioID proximity labeling results and supporting a role for KLF4 in regulating *PSA* transcription, as suggested by our luciferase *PSA* reporter assays (Fig. 3*A*). We further found that in KLF4-depleted cells, DHT stimulation increased AR-driven luciferase reporter signal by at least 1.7-fold, while cells depleted of AR displayed a 3.7-fold decrease (*p* ≤ 0.0001) (Fig. 3, *C* and *D*). Interestingly, KLF4 was previously reported to directly bind to the *AR* promoter and KLF4 depletion to decrease AR protein levels in LNCaP cells, which express a mutated AR (58). To determine whether KLF4 knockdown affected endogenous wild-type *AR* expression in LAPC4 cells, we analyzed mRNA levels *via* RT-qPCR. We found that *AR* RNA levels were unchanged in KLF4-depleted cells relative to controls, both in nonstimulated and in DHT-stimulated cells (Fig. 3*E*). We also observed that AR protein expression was unaffected (Fig. 3*C*). Consistent with this observation, ectopic 3xFLAG-KLF4 expression did not lead to increased AR levels (Fig. 3*G*). We detected a slight increase in *KLF4* expression in AR-depleted cells with one of the two siRNAs targeting *AR* (Fig. 3*F*); however, this was not confirmed at the protein level (Fig. 3*C*).

To confirm that the *PSA* reporter repression was due to KLF4 loss-of-function, we re-expressed 3xFLAG-tagged KLF4 that is not affected by siRNAs, which both target the 3’UTR sequence. Re-expression of 3xFLAG-KLF4, but not 3xFLAG-BirA∗, decreased luciferase levels to those of control LAPC4 cells in the presence of DHT (Fig. 3, *G* and *H*). Cells transfected with siCtrl or siAR did not display a change in luciferase levels following 3xFLAG-KLF4 re-expression compared with controls. To corroborate our findings in another prostate cell line that expresses wild-type AR, we opted for VCaP cells (59). We confirmed that AR-dependent transcription of the luciferase transgene was increased following DHT stimulation and that it was blocked in AR-depleted cells. Moreover, we showed that KLF4 knockdown led to a strong augmentation of luciferase levels, thus supporting the observations made in LAPC4 cells (Fig. 3, *I* and *J*). Taken together, our results suggest that AR and KLF4 are functional partners.

**Fig. 4:**
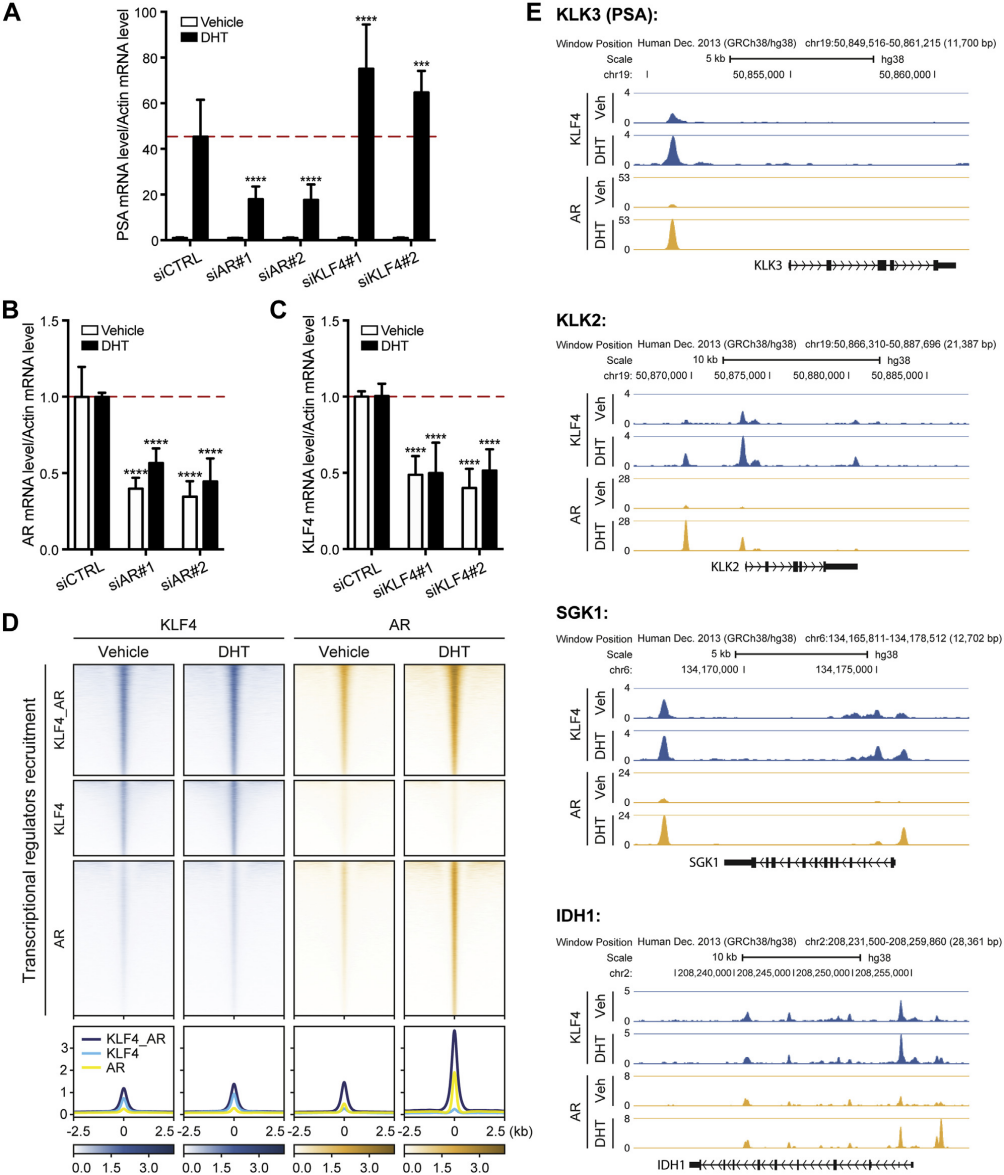
**A large fraction of KLF4 colocalizes with AR in the genome and acts as a repressor of the AR target gene *KLK3* (*PSA*) in LAPC4 cells.***A–C*, control, siAR, or siKLF4 transfected LAPC4 cells were stimulated with a vehicle or 10 nM DHT, and RT-qPCR was performed to assess PSA mRNA levels (*A*). AR (*B*) and KLF4 (*C*) depletion efficiency was also determined by RT-qPCR. Data was normalized to actin mRNA levels, and then to the control condition to obtain relative values. Mean values and standard deviation from three independent experiments are presented (∗∗∗*p* ≤ 0.001; ∗∗∗∗*p* ≤ 0.0001). *D*, heat map showing the co-occupancy of KLF4 and AR across the genome of LAPC4 cells. *Top*: Density heat maps representing KLF4 and AR ChIP-seq intensities in the absence (control) or presence of DHT at three groups of genomics regions (occupied by AR and KLF4 (n = 35,709), occupied by KLF4 only (n = 24,190), and occupied by AR only (n = 49,877)). Regions were ranked according to the total read density in the KLF4 control condition. Color scales indicate bins per million mapped reads (BPM). Bottom: Average read density plots for the same groups of regions. A region of 5kb centered on the occupied region is displayed. *E*, ChIP-Seq occupancy profiles of KLF4 and AR in control and DHT-stimulated cells. Genome tracks show colocalization of KLF4 (*blue*) and AR (*yellow*) ChIP-Seq peaks in the neighborhood of *KLK3* (*PSA*), *KLK2*, *SGK1*, and *IDH1* genes that are known to be regulated by AR. ChIP-Seq profiles are displayed in BPM. Gene depictions are presented below the gene tracks.

To validate that KLF4 acts as a repressor of the AR target gene *PSA* in an endogenous context, we performed RT-qPCR analyses of mRNA levels of *PSA*. Following DHT stimulation, *PSA* mRNA levels increased 45-fold in LAPC4 cells. This activation was hindered in AR-depleted cells, as *PSA* messengers decreased by 2.5-fold (*p* ≤ 0.0001, two-way ANOVA) (Fig. 4, *A* and *B*). As observed in the luciferase reporter assay, *PSA* mRNA increased significantly by an average of 1.5-fold (*p* ≤ 0.0001, two-way ANOVA), following KLF4 knockdown with two independent siRNA sequences tested (Fig. 4, *A and C*). This latter increase in *PSA* expression was not due to an accumulation of AR in the nucleus (supplemental Fig. 2). Together, our data strongly support the idea that KLF4 acts as a repressor during androgen-dependent *PSA* gene activation.

To determine if AR and KLF4 shared cis-regulatory regions genome-wide, we surveyed their recruitment with/without a 1h DHT treatment using chromatin immunoprecipitation coupled with massively parallel DNA sequencing (ChIP-seq) in LAPC4 cells. As expected, the number of regions occupied by AR increased from 24,379 in control conditions to 85,312 when cells were stimulated with DHT. For KLF4, 26,891 regions were observed in control cells compared with 59,806 in DHT-stimulated cells (supplemental Table S3 and supplemental Fig. S3). Overall, when we considered the entire set of regions occupied by KLF4 and/or AR in all conditions, 33% were bound by both transcription factors (Fig. 4*D*). Interestingly, for regions co-occupied by KLF4 and AR, the signal densities for both transcription factors were correlated, thus supporting co-occupancy. In addition, as expected, signal density for AR increased following DHT stimulation while KLF4 was relatively stable. While many regions occupied by the AR following the DHT treatment were also occupied by KLF4 in control conditions, a similar fraction was not occupied (supplemental Fig. S3), suggesting different models for interactions between these two transcription factors. In total, we found 4097 genes with KLF4 and AR both binding at their promoter region following DHT stimulation (supplemental Table S4). Closer examination of density profiles of well-known AR target genes supported co-occupancy. For example, the well-characterized enhancer region of the PSA gene (*KLK3*) was occupied by both KLF4 and AR (Fig. 4*E*). Similar results were observed for *KLK2*, *SGK1*, and *IDH1*. Therefore, the ChIP-seq experiments support subnuclear colocalization of KLF4 and AR, suggesting a functional association.

## Discussion

In this work, we report a high-confidence proximity network for AR in prostate cells. We found that the majority of network components associate with AR in a ligand-dependent manner. Strikingly, we further reveal 213 new proteins associated with AR. Recently, Lampiäinen *et al.* reported an AR proximity network comprised of 32 preys in HEK293 cells (20). We identified 24/32 proteins that these authors revealed in DHT-stimulated cells, as well as 237 additional DHT-dependent interactions. Discrepancies are likely attributed to the differences in cell lines used in the two studies, as well as biotin/DHT treatment durations, composition of controls for BioID experiments, and MS data analysis workflows. We propose that the higher complexity of the AR proximity network that we reveal here is due to higher endogenous expression of wild-type AR and responsiveness to androgens of LAPC4 versus HEK293 cells, as suggested by previous reports (21).

The AR proximity network that we uncovered outlines associations between AR and core components of the transcriptional and chromatin remodeling machineries, in addition to highlighting associations with proteins implicated in RNA processing, DNA metabolism, or DNA repair. For example, we identified 109 transcriptional cofactors, including components of the mediator complex and the NCoR complex. Moreover, we found a number of proteins previously described to be part of chromatin remodeling complexes such as BAF and NuRD. Many new interactors are subunits of protein complexes previously reported to associate with AR. This observation emphasizes the potential of BioID proximity labeling to identify proteins present in the vicinity of a given bait.

Of note, many interactors of AR found in the BioGRID, GSP-Prot, or IntAct database were not determined as high-confidence interactors in our analysis (supplemental Table S1), highlighting limitations of the BioID proximity labeling approach. For example, HSP90AA1 (Heat shock protein HSP90-alpha), HSP90AB1 (Heat shock protein 90-beta), HSPA1B (Heat shock 70 kDa protein 1B), HSPA2 (Heat shock-related 70 kDa protein 2), HSPA5 (Heat shock 70 kDa protein 5), and HSPD1 (60 kDa heat shock protein) were detected as AR proximity interactors, but they did not pass the high-confidence threshold due to their presence in the 3xFLAG-BirA∗ controls (supplemental Table S1).

Using an affinity purification approach, we confirmed the previously reported association between AR and MED1 or KMT2A, but we also validated new interaction partners for AR (Fig. 3*B* and supplemental Fig. 1, *B–G*). Among those, we identified KLF4 as a new AR-associated protein. Interestingly, Siu *et al.* previously discovered the existence in LNCaP cells of a reciprocal feedback loop between KLF4 and AR, in which each protein binds to the promoter of each other’s gene (58). This observation was confirmed in our ChIP-Seq data. However, in LAPC4 cells, we did not observe a change in *AR* expression or protein levels upon KLF4 knockdown, neither a change in *KLF4* expression or protein levels in cells depleted of AR. Therefore, it remains possible that the self-reinforcing loop between KLF4 and AR is cell-type specific. However, we did observe a significant overlap genome-wide between regions occupied by KLF4 and AR. These observations underline the importance of further examining AR-associated proteins in several PCa cell lines harboring different genetic and proteomic landscapes to possibly extend findings to primary PCa.

KLF4 function in transcriptional regulation is considered to be context-specific (60, 61). For example, during induced reprogramming of somatic cells into pluripotent cells, KLF4 represses somatic genes in an early phase and subsequently activates pluripotency genes (62). Consistent with this latter role, we show that KLF4 represses the transcription of the AR target gene *PSA*, but exclusively yet consistently when cells are stimulated with DHT to activate AR. Interestingly, KLF4 was previously reported to directly associate with the DNA-binding region of ERα, thereby inhibiting the transcriptional activity of this nuclear receptor, in an estrogen-dependent manner (63). Our data with AR suggests a common mechanism for the regulation of nuclear receptor-dependent *PSA* gene regulation by KLF4.

Recently, Fei *et al.* reported a genome-wide CRISPR/Cas9 knockout screen to identify essential genes in LNCaP PCa cells (64). Among the top 1000 gene products (including AR) that they deemed essential, we identified 15 in our AR BioID experiments (supplemental Table S2). Interestingly, comparison of pathway information revealed a number of common functional groups (Fig. 2), including RNA processing, transcription initiation, and DNA replication. While the proteome landscape between LNCaP cells, which feature a mutated AR, and LAPC4 cells utilized in the BioID experiments may be different, the relatively low number of essential genes among the AR proximity interaction landscape suggests that most candidates could be successfully targeted to modulate AR activity in PCa cells and suggests novel possibilities of treatment for prostate cancer patients.

KLF4 has been associated with either oncogenic properties, such as in osteosarcoma (65), either with tumor suppressive functions, such as in the lung (66), gastric (66), and prostate cancers (67). For example, overexpression of KLF4 in T24 urothelial bladder carcinoma cells leads to p21 accumulation, G1-phase arrest, and a significant decrease of tumor growth in a xenograft model (68). It was also reported to decrease proliferation of colorectal cancer cells by repressing cyclin D1 transcription (69). In prostate cancer, *KLF4* transcription and KLF4 protein levels were decreased in metastases, while its re-expression inhibited prostate cancer cell migration and invasion (67). This suggested its potential usage as a prognosis marker. Our work proposing that KLF4 can impair AR adds another layer of complexity to KLF4 tumor suppressive function in controlling prostatic cell proliferation and survival. Moreover, this leads to the premise that simultaneous downregulation of KLF4 with increased AR levels could indicate a less favorable prostate cancer prognosis.

Together, we provide a large, high-confidence proximity interaction network for AR obtained from androgen-responsive cells. We further demonstrate the relevance of our data by characterizing the codistribution of KLF4 and AR across the genome, as well as a key repressive function for KLF4 in the regulation of the AR target gene *PSA*.

## Data Availability

The mass spectrometry proteomics data have been deposited to the ProteomeXchange Consortium *via* the PRIDE partner repository with the data set identifiers PXD011974 and 10.6019/PXD011974 (70).

The sequencing data generated for this publication is available on GEO (71), under accession number GSE161189.

## Supplementary data

This article contains supplemental data.

## Conflict of interest

The authors declare no competing interests.
