# Supplementary material for: Proximity-dependent Mapping of the Androgen Receptor Identifies Kruppel-like Factor 4 as a Functional Partner
Source: Mol Cell Proteomics. 2021 Feb 26;20:100064. doi: 10.1016/j.mcpro.2021.100064 (PMC8050775; doi:10.1016/j.mcpro.2021.100064)
Supplement: Supplemental Figures and Table legend [file mmc1.pdf]

SUPPLEMENTAL DATA

Proximity-dependent mapping of the Androgen Receptor identifies Kruppel-Like Factor 4 as a functional partner.

Lauriane Vélot[1,2,3], Frédéric Lessard[1,2,3], Félix-Antoine Bérubé-Simard[1,2], Christophe Tav[1,2,4], Bertrand Neveu[1,2], Valentine Teyssier[1,2,3], Imène Boudaoud[1,2], Ugo Dionne[1,2,3], Noémie Lavoie[1,2,3], Steve Bilodeau[1,2,4,6], Frédéric Pouliot[1,2,5*] and Nicolas Bisson[1,2,3,6*]

[1] Centre de recherche du Centre Hospitalier Universitaire (CHU) de Québec-Université Laval, Axe Oncologie, Québec, QC, Canada

[2] Centre de recherche sur le cancer de l'Université Laval, Québec, QC, Canada

[3] PROTEO-Quebec Network for Research on Protein Function, Engineering, and Applications, Québec, QC, Canada

[4] Centre de recherche en données massives de l'Université Laval, Québec, Québec, Canada

[5] Department of Surgery, Faculté de Médecine, Université Laval, Québec, QC, Canada

[6] Department of Molecular Biology, Medical Biochemistry and Pathology, Faculté de Médecine, Université Laval, Québec, QC, Canada.

* To whom correspondence should be addressed: frederic.pouliot@crchudequebec.ulaval.ca, nick.bisson@crchudequebec.ulaval.ca

RUNNING TITLE: AR proximity network reveals KLF4 as a functional partner.

CONTENTS

SUPPLEMENTAL FIGURES

Suppl. Figure S1: Validation of AR BioID experiments

Suppl. Figure S2: KLF4 does not increase AR localization to the nucleus

Suppl. Figure S3: Distribution of AR and KLF4 enriched regions in control and DHT conditions

SUPPLEMENTAL TABLES LIST (EXCEL FILES)

Table S1. List of BioID identified peptides and proteins, as well as calculated SAINT scores.

Table S2. List of essential genes in LNCaP PCa cells (according to Fei et al., PMID 28611215) found in the BioID experiments and presented in Figure 2.

Table S3. Genomic coordinates of KLF4 and AR binding sites in control and DHT conditions.

Table S4. Annotation of genes with binding of KLF4 and AR in control and DHT conditions at promoters (downstream: 1kb, upstream: 5kb)

DATA AVAILABILITY

The mass spectrometry proteomics data have been deposited to the ProteomeXchange Consortium via the PRIDE partner repository with the dataset identifiers PXD011974 and 10.6019/PXD011974.

The sequencing data generated for this publication will be available on GEO, under accession number GSE161189.

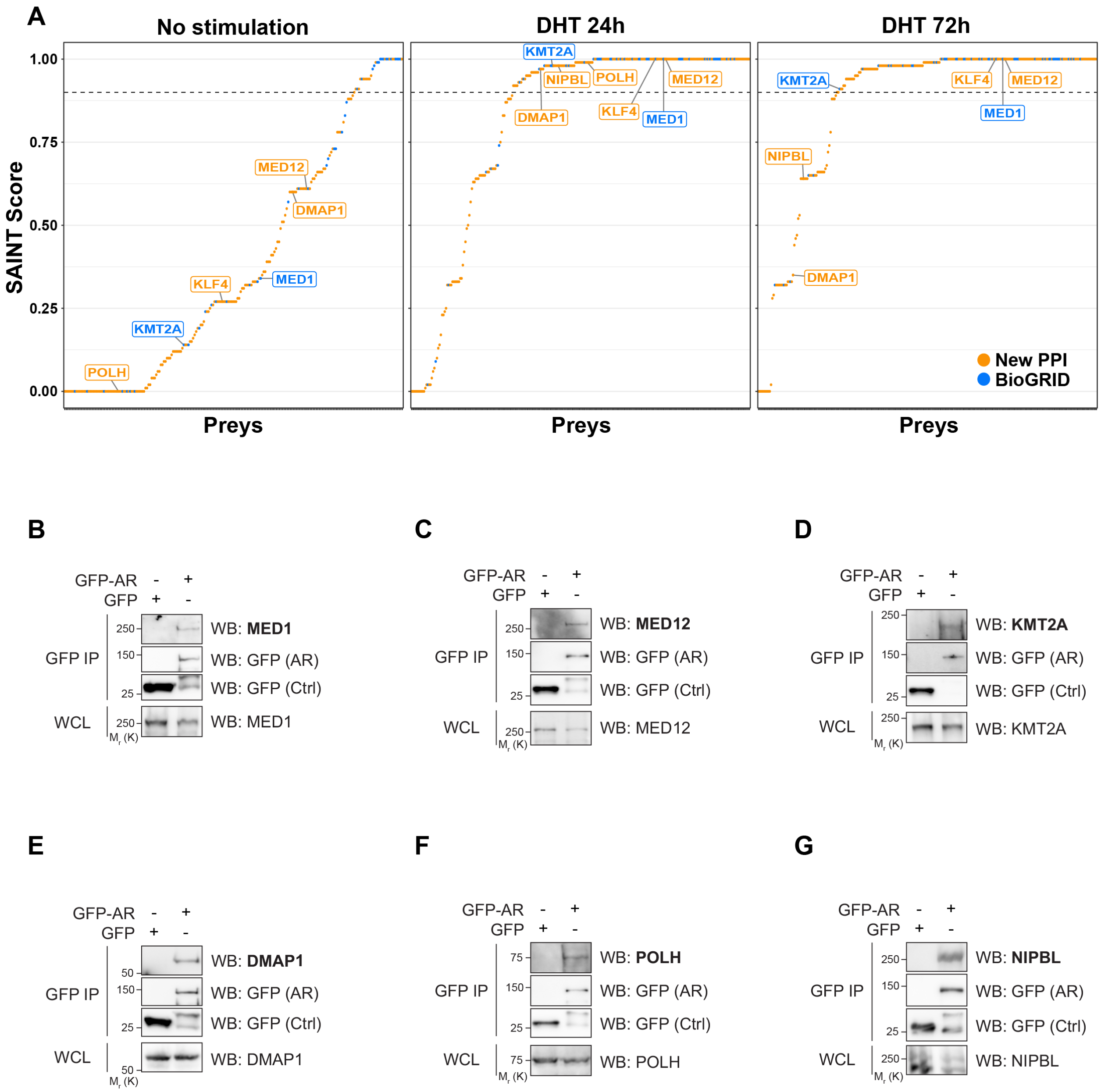

Suppl. Figure S1: Validation of AR BioID experiments

(A) SAINT score distribution for each of the 268 AR proximity partners detected in the BioID experiments with non-stimulated cells (left), 24h (center) or 72h (right) DHT stimulation. The high-confidence 0.9 SAINT threshold (~1% FDR) is represented as a horizontal dashed line. Blue preys previously reported as AR partners in the BioGRID database are displayed, as well as AR interactors further validated in Figure 3B and supplemental Figure 1B-G. (B-G) Western Blot analysis of endogenous MED1 (B), MED12 (C), KMT2A (D), DMAP1 (E), POLH (F) and NIPBL (G) following GFP affinity purification in HEK293T cells transfected with GFP/AR-GFP. Blots representative of 2 independent experiments are displayed.

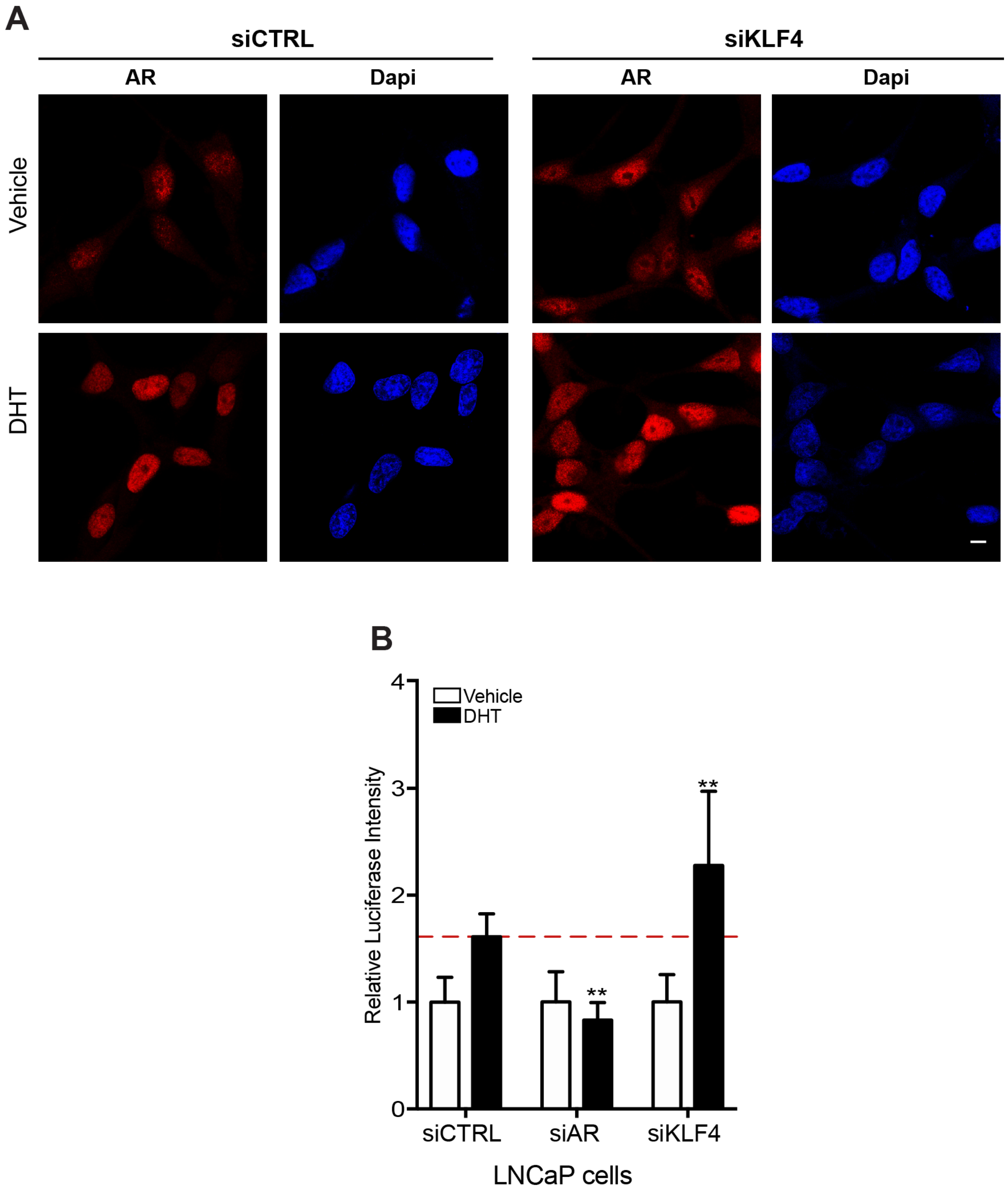

Suppl. Figure S2: KLF4 does not increase AR localization to the nucleus

(A) Control, siAR or siKLF4 transfected LNCaP were stimulated with a vehicle or 10 nM DHT and analyzed by immunofluorescence for AR (red) and DAPI (blue) to assess AR localization. Representative images are presented (scale bar: 10 µm). (B) The same cell lines were infected with a luciferase reporter gene coupled with a *PSA* promoter. Luciferase activity was normalized to the total protein amount for each sample, and to the vehicle-treated condition to obtain relative luciferase intensities for each sample. Mean values and standard deviation from three independent experiments are presented (** $p \leq 0.01$).

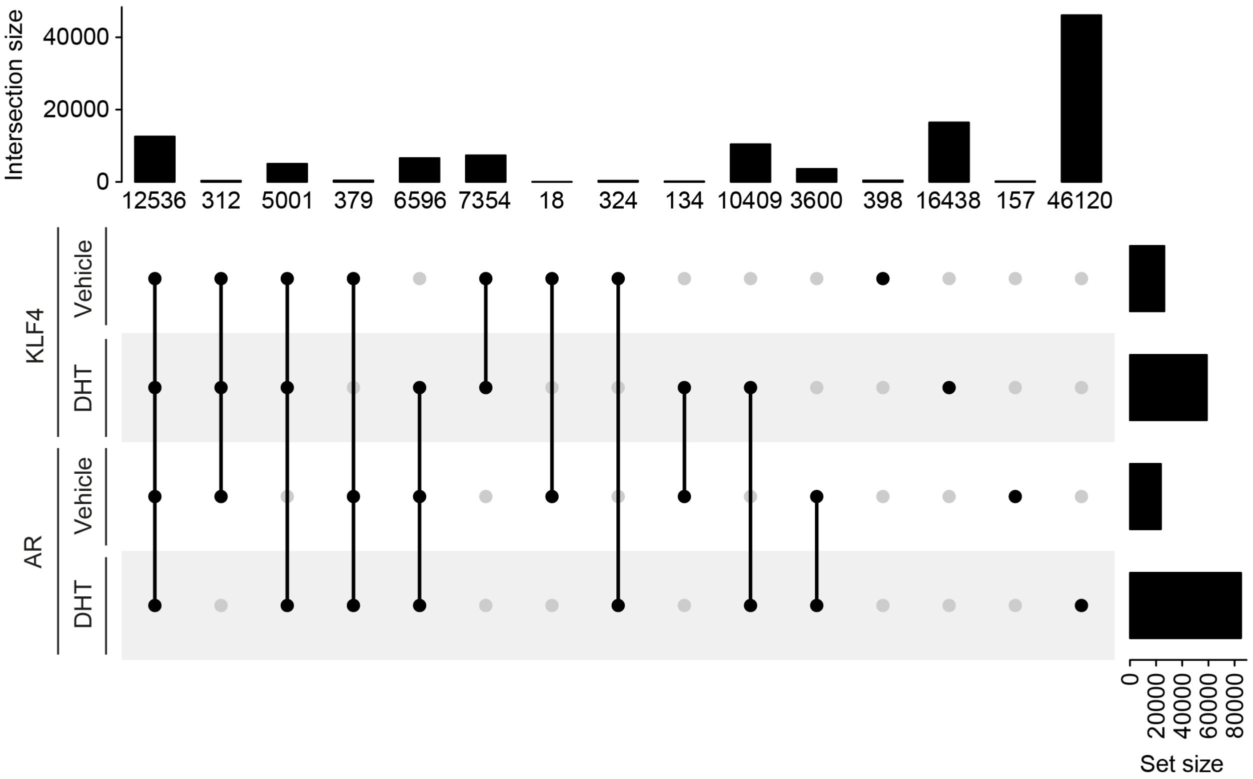

Suppl. Figure S3: Distribution of AR and KLF4 enriched regions in control and DHT conditions

Upset plot showing the overlap between enriched regions of both transcription factors (KLF4 and AR) in control and DHT conditions. The abscissae represent the total number of genomic regions for each group while the ordinates report the number of overlaps.
